# A proposal for changes to the European Union syphilis surveillance case definition using evidence from evaluations in Ireland

**DOI:** 10.2807/1560-7917.ES.2019.24.45.1900311

**Published:** 2019-11-07

**Authors:** Naomi Petty-Saphon, Melissa Brady, Gillian Cullen, Fionnuala Cooney, Phil Downes, Sarah Doyle, Paul Holder, Fiona Lyons, Derval Igoe

**Affiliations:** 1 Health Protection Surveillance Centre, Dublin, Ireland; 2 Department of Public Health, HSE-East, Dublin, Ireland; 3 Department of Public Health, HSE South East, Kilkenny, Ireland; 4 National Virus Reference Laboratory, Dublin, Ireland; 5 St James’s Hospital, Dublin, Ireland

**Keywords:** syphilis, surveillance, Ireland, case definition

## Abstract

Syphilis remains a disease of public health importance, with considerable health effects if not treated. Concurrent infection with syphilis and untreated HIV facilitates HIV transmission. The incidence of syphilis in Europe has been increasing, particularly among men who have sex with men (MSM) and in MSM with HIV. However, there is heterogeneity among countries in the case definition used for syphilis and in reported syphilis notification rates. In Ireland, we have undertaken a number of refinements of the national syphilis surveillance system since 2014, including refinement of the laboratory thresholds for notification (rapid plasma reagin 1:16 and/or positive IgM). This article outlines the steps taken and some of the challenges we faced. Our current case definition now accurately reflects the epidemiology of syphilis in Ireland and our current surveillance provides timely information for action, while not reducing the sensitivity of the system too much. For countries where surveillance is driven mainly by laboratory reporting and where obtaining clinical details is challenging, these thresholds for notification may be a pragmatic solution.

## Background

Despite availability of sensitive diagnostic tests and effective treatment, syphilis remains a serious health problem, both for individuals and for public health [1]. Without treatment, infection may progress and lead to serious, potentially long-term, health consequences. There are several stages of syphilis infection: Primary syphilis usually involves one lesion that occurs at the site of infection. These lesions are painless and may be in sites that are not visible, therefore may be unnoticed or not recognised. Secondary syphilis occurs 4–8 weeks after primary syphilis; it involves a systemic infection with bacteraemia, symptoms include a widespread rash and wart-like lesions called condylomata lata [2]. Latent syphilis is an asymptomatic infection and is divided into early latent syphilis (infection of less than 12 months duration) and late latent syphilis (infection of more than 12 months duration). While syphilis can be treated with antibiotic therapy, no immunity develops following infection and successful treatment and re-infection can occur with subsequent exposure [3].

Syphilis reporting aims to distinguish between the stages of disease in order to identify new and potentially infectious cases so that public health action can be taken. However, this can be complicated; the primary and secondary stage may only be detected through serological testing, and it may not be possible to identify how long a latent infection has been present (infections present for less than 12 months are notifiable in Ireland as they are infectious, whereas infections present for more than 12 months are not [4]). Only early syphilis (ES) cases, i.e. primary, secondary and early latent, are infectious [5] and are of public health importance. High quality and accurate reporting of infectious syphilis is needed for analysis of trends and in order to gather information for relevant and timely control activities. The aim of this paper is to describe the challenges and some of the steps taken to improve syphilis surveillance in Ireland.

## The incidence of syphilis

The incidence of syphilis has been increasing in Europe and other high-income countries (for example in the United States, Canada and Australia) in recent years [6,7]. The highest incidence of syphilis is in men; cases in the European Union and European Economic Area (EU/EAA) have been increasing since 2010 and by 2016 had reached the highest rates reported since EU/EAA surveillance began [7]. This increase has largely been seen among men who have sex with men (MSM) [6], among whom rates in the EU/EAA increased by 164% between 2010 and 2016 [7].

However, there is heterogeneity in trends between countries; rates in eastern EU/EAA countries peaked in the 1990s and early 2000s at very high rates and then declined, whereas the rates in western and central EU/EAA countries started from low levels in the early 2000s and have been steadily increasing [7]. Although rates in women are low, some western EU/EAA countries have also seen increases in rates of syphilis among women, which is of concern because of the risk of mother-to-child transmission of syphilis [7]. In 2016 the average rate of syphilis in the EU/EAA was 6.1 cases per 100,000 population (this ranged from fewer than two cases per 100,000 population to 9.9 cases per 100,000 population). In Ireland, the reported rate of syphilis in 2016 was close to the EU/EAA average at 6.2 cases per 100,000 population [6], but has since increased to 8.4 per 100,000 population in 2017 [8]. Further complicating a comparison of trends between countries is the fact that different countries have included different definitions of syphilis in their total syphilis reporting, with some countries including syphilis infection of any stage and some including only early infections [9].

While it is expected that there will be variation between the types of surveillance systems and information used for surveillance in different countries, EU countries are expected to use the EU case definition [4].

Many EU countries, including Ireland, were not using the EU-2012 syphilis case definition (current at the time we reviewed our case definition) for surveillance. Information on what definition countries use is available as part of the information reported by countries to the European Centre for Disease Prevention and Control (ECDC) using The European Surveillance System (TESSy). For example in 2016, three different EU case definitions were in use: EU-2002 (2 countries), EU-2008 (7 countries) and EU-2012 (9 countries), and for 10 countries, the case definition used was either unknown, not specified or classified as other [9]. The accuracy of these data may also be questioned, as at least in the case of Ireland, the changes in the Irish case definition were not fully logged in TESSy. 

To have one standard syphilis case definition used by all countries in the EU would be ideal as the current variation complicates interpretation of surveillance data at an EU level. The recently amended EU-2018 case definition from June 2018 [4] addresses some of the issues highlighted in this review, but not all. For operational reasons, there have been challenges in obtaining timely information on ES in Ireland, prompting a focus in recent years on improving timeliness of information, while also trying to simplify and shorten reporting periods. Some of the lessons learnt from this process as described in this paper may be applicable to other countries.

## Syphilis surveillance in Ireland

In Ireland, syphilis has been notifiable since 1948 [10] and case-based data on syphilis cases have been collected since 2000. All clinicians and clinical directors of laboratories have a statutory obligation to notify all cases of syphilis as per the Irish case definition [11]. Over time, evaluations of syphilis surveillance (excluding congenital syphilis) have highlighted a number of challenges, resulting in a number of changes.

## Changes to the laboratory criteria

Since 2013, all laboratories have uploaded syphilis test results that meet the laboratory case definition criteria for notification to the Computerised Infectious Disease Reporting (CIDR) system, the national information system for the statutory surveillance of notifiable infectious diseases in Ireland. Before 2014, the case definition for syphilis followed the EU case definition, EU-2012. However, some ambiguities in the use of this case definition in Ireland were identified which limited its capacity to accurately measure ES in the Irish context. For example, the case definition aimed to detect infectious syphilis cases; however, it was found that laboratories were notifying public health of syphilis serology that was likely to reflect previously treated or late latent infection (i.e. a latent infection for more than 12 months). This meant that the laboratory criteria for the case definition were not specific enough for ES and there was a reliance on a case being clinically confirmed as ES by a clinician to be included in ES surveillance. 

In 2013, when the surveillance system relied on the enhanced surveillance data from clinicians to confirm the stage of infection, there were 545 syphilis notifications in Ireland, but only 185 (34%) were specified to be ES and 176 (33%) were not ES. Importantly, the stage was not specified for 193 cases (35%); these cases may or may not have been ES cases, but the clinical data were missing. This highlighted a need to revise the case definition in Ireland to improve our data quality. In addition, some of the laboratory requirements included in the case definition were not usual practice in laboratory diagnosis in Ireland; the case definition at that time included confirmatory IgM testing using an alternative assay (IgM enzyme immunoassay or IgM immunoblot), which was not usually performed, and did not include tests in use, such as rapid plasma reagin (RPR). 

To reflect practice in Ireland, the following changes to the laboratory criteria in the case definition were made: confirmation by a second IgM assay was not necessary, the criteria instead made reference to treponemal and non-treponemal antibodies which allowed for different combinations of screening and confirmatory tests and chemiluminescence immunoassay and cardiolipin non-Tp (RPR and venereal disease research laboratory test) were added to the laboratory criteria. In Ireland, where only a few laboratories provide full syphilis serology testing, repeat samples are often tested in the same laboratory; therefore a sample history for a patient is often available. These are used to identify re-infections, and syphilis re-infections were notified as per the laboratory’s own criteria. Following the changes to the laboratory criteria, all laboratory-notified cases were classified as confirmed ES cases and only de-notified if subsequently identified not to be ES by clinical services. It was thought that these changes would better identify ES cases, provide early indication of changing trends, enable timely response and considerably reduce the number of late (non-notifiable) cases reported to public health departments who were tasked with seeking the stage of infection from clinical services on all syphilis cases notified by laboratories. 

At the time when we reviewed our case definitions, the EU-2012 case definition was in use; it was amended in 2018 and the need for confirmatory IgM was removed and late latent cases of syphilis are no longer under EU surveillance. As a result, the Irish case definition is more similar to the EU definition, however our laboratory thresholds for notification differ, as discussed below.

## Review and further revision of the laboratory criteria

A review of the impact of the change in notification criteria was carried out in 2015 and 2016 in the Health Service Executive (HSE)-East area in Ireland for all notifications received in the first quarter of 2014. This review categorised notifications into infectious cases (i.e. ES) and de-notified cases (those that did not meet the case definition). Of 74 cases notified by laboratories between January and April, 33 were subsequently de-notified as they were clinically determined not to be infectious syphilis. Not only was this a resource-intensive activity for clinical services and public health departments but there were also considerable delays from the time of laboratory notification to final clarification of the status of cases and interpretation of trends. As part of the review, to assess laboratory indicators of syphilis infectivity, we categorised cases by IgM and RPR result. This assessment found that 29 of 32 IgM-positive cases were infectious and 30 of 42 de-notified cases had a negative IgM result and an RPR of ≤ 1:8 (Figure) [12].

**Figure:**
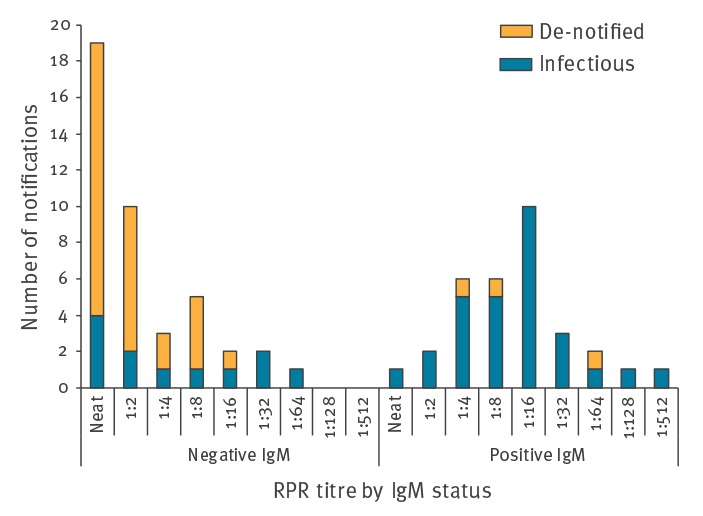
Syphilis notifications (infectious and de-notified cases), HSE-East, Ireland, January–April 2014 (n = 74)

HSE: Health Service Executive; RPR: rapid plasma reagin.

This suggested that a positive IgM test or an RPR titre of at least 1:16 were appropriate measures of syphilis infectivity. Although this review only included cases notified for one quarter of a year, we thought that the pattern of these notifications were in line with the patterns seen throughout previous years, and in order to collect accurate and representative information, further changes were required at this point. Based on this review, the laboratory criteria for notifying new syphilis cases were further refined in 2016 [13]. All notified cases were assumed to be ES unless stated otherwise but the laboratory RPR threshold for notification were raised to a titre of at least 1:16. The rationale for these changes to the case definition were that the new criteria would lead to fewer non-ES cases being reported and less de-notifications at a later stage. We thought that the changes would also simplify the notification process and enable provision of more timely information for surveillance purposes. It was acknowledged that this revision to the laboratory criteria would result in some cases not being detected by the surveillance system. But based on the number of cases that did not meet the new criteria in the review (i.e. IgM-negative cases with an RPR < 1:16), this proportion was thought to be low (ca 4%). At that time, there was a national outbreak of HIV and syphilis among MSM, and timeliness of the surveillance system was considered more important than small reductions in sensitivity. 

The EU case definitions (2012 and 2018) and the 2016 Irish case definition are displayed in the Table.

**Table:** Syphilis case definitions, European Union and Ireland

EU case definition (2012)	EU case definition (2018)	Irish case definition (2016)
Clinical criteria		
Primary syphilis		
Any person with one or several (usually painless) chancres in the genital, perineal or anal area, mouth, pharyngeal mucosa or other extragenital area	Any person with one or several (usually painless) chancres in the genital, perineal or anal area, mouth, pharyngeal mucosa or other extragenital area	Any person with one or several (usually painless) chancres in the genital, perineal or anal area, mouth, pharyngeal mucosa or other area
Secondary syphilis		
Any person with at least one of the following five: - Diffuse maculopapular rash, often involving palms and soles - Generalised lymphadenopathy - Condyloma lata - Enanthema - Diffuse alopecia	Any person with at least one of the following five: - Diffuse maculopapular rash often involving palms and soles - Generalised lymphadenopathy - Condyloma lata - Enanthema - Diffuse alopecia	Any person with at least one of the following: - Diffuse maculopapular rash often involving palms and soles - Generalised lymphadenopathy - Condyloma lata - Enanthema - Alopecia diffusa - Ocular manifestations of early syphilis - Neurological manifestations of early syphilis
Early latent syphilis (< 1 year)		
A history of symptoms compatible with those of the earlier stages of syphilis within the previous 12 months	No symptoms and a history of symptoms compatible with those of the earlier stages of syphilis within the previous 12 months. Note that ocular and neurological manifestations may occur at any stage of syphilis.	Positive syphilis serology, no symptoms or signs of early syphilis and a negative reference screening test for syphilis within the previous 12 months. Note that a case may be asymptomatic.
Late latent syphilis (> 1 year)		
Any person meeting laboratory criteria (specific serological tests)	Not under EU/EEA surveillance	Not under Irish surveillance; if notified, cases are subsequently de-notified. In Ireland, all cases under surveillance are classified as early syphilis.
Laboratory criteria		
At least one of the following four laboratory tests: - Demonstration of *Treponema pallidum* in lesion exudates or tissues by dark-field microscopic examination - Demonstration of *T. pallidum* in lesion exudates or tissues by DFA test - Demonstration of *T. pallidum* in lesion exudates or tissues by PCR - Detection of *T. pallidum* antibodies by screening test (TPHA, TPPA or EIA) and additionally detection of *T. pallidum* IgM antibodies (by IgM-ELISA, IgM immunoblot or 19S-IgM-FTA antibodies) confirmed by a second IgM assay	At least one of the following: - Demonstration of *T. pallidum* in lesion exudates or tissues by dark-field microscopic examination - Demonstration of *T. pallidum* in lesion exudates or tissues by DFA test - Demonstration of *T. pallidum* in lesion exudates or tissues by NAAT - Detection of* T. pallidum* antibodies by screening test (TPHA, TPPA or EIA) and additionally detection of either *T. pallidum* IgM antibodies (e.g. IgM-ELISA or immunoblot or 19S-IgM-FTA antibodies) or non-*T. pallidum* antibodies (e.g. RPR, VDRL)	New infections with at least one of the following laboratory tests: - Demonstration of *T. pallidum* in appropriate lesions, exudates or tissues by dark-ground microscopic examination - Demonstration of* T. pallidum* in appropriate lesions, exudates or tissues by PCR - Detection of *T. pallidum* antibodies (total antibodies) using EIA and TPHA/TPPA and additionally detection of *T. pallidum* IgM antibodies (e.g. IgM ELISA or immunoblot or 195-IgM-FTA antibodies) - Detection of *T. pallidum* antibodies (total antibodies) using EIA and TPHA/TPPA and additionally detection of cardiolipin non-*T. pallidum* IgM with RPR titre ≥ 1:16 For re-infections, laboratories should use their own criteria
Epidemiological criteria		
Primary/secondary syphilis		
An epidemiological link by human-to-human (sexual contact).	An epidemiological link by human-to-human (sexual contact)	NA
Early latent syphilis (< 1 year)		
An epidemiological link by human-to-human (sexual) contact within the 12 previous months	An epidemiological link by human-to-human (sexual) contact within the 12 previous months	NA
Case classification		
Possible case		
NA	NA	NA
Probable case		
Any person meeting the clinical criteria and with an epidemiological link	Any person meeting the clinical criteria and with an epidemiological link	NA
Confirmed case		
Any person meeting the laboratory criteria for case confirmation	Any person meeting the laboratory criteria for case confirmation	Any person meeting the clinical criteria for early syphilis and the laboratory criteria for case confirmation

DFA: direct fluorescent antibody; EU: European Union; EEA: European Economic Area; EIA: enzyme immunoassay ELISA: enzyme-linked immunosorbent assay; FTA: fluorescent treponemal antibody-absorption test; NA: not applicable; NAAT: nuclear acid amplification techniques; RPR: rapid plasma reagin; TPHA: *T. pallidum *haemagglutination assay; TPPA: *T. pallidum* particle agglutination; VDRL: venereal disease research laboratory test.

## Evaluation of case definitions

To examine the effect of the changes to the case definitions used in Ireland, an evaluation of the sensitivity, completeness and timeliness of the syphilis surveillance system was undertaken in 2018. In this evaluation, sensitivity was considered to be the ability of the surveillance system to detect a health event; at the level of case reporting, sensitivity refers to the proportion of cases of a disease detected by the system [14]. Because other prevalence data for syphilis are not available in Ireland, the evaluation quantified the number of ES notifications the system detected for each case definition. It was not possible to include cases that were not reported. This evaluation identified that the changes have improved the syphilis surveillance system in Ireland. However, syphilis rates have increased in Ireland and internationally [6] and when interpreting sensitivity, it should be noted that changes in sensitivity may represent true changes in syphilis rates in the population rather than, or in addition to, changes to the system. 

The first change to the case definition in 2014 increased the sensitivity of the system but was not timely in determining ES cases quickly and led to a large burden of inappropriate work in terms of follow up of cases many of which were subsequently found not to be cases of ES. There were 1,102 cases of syphilis notified in HSE-East in that time (1 January 2014–30 June 2016; 30 months), of which 415 were subsequently de-notified. The change to the laboratory criteria in 2016 decreased the sensitivity of the system, which was successful in detecting ES cases early, and reducing the need to follow up cases which were subsequently de-notified. There were 662 notifications in the same health region in that time (1 July 2016–30 June 2018; 24 months), 24 of which were subsequently de-notified. This indicates that the proportion of cases requiring follow-up but that were not cases of ES reduced from 38% to 4% with the revision to the laboratory criteria. This indicates that the current laboratory thresholds for notification are appropriate. Fifteen of 24 de-notified cases were de-notified as they were staged as late latent syphilis, other reasons for de-notification included notifications in error or case duplication.

The time of importance for public health is the interval between the diagnosis of ES and when public health authorities are notified via the electronic reporting system, CIDR. While not all patients are symptomatic, the time between the onset of symptoms to knowing that the case is ES is also of importance; this will be affected by external factors to the surveillance system such as patient recognition of symptoms and access to healthcare services. The changes to the case definition have increased the timeliness of the system; there was a decrease in the median and spread of time from laboratory test to public health awareness of an ES case. In 2013, this median was 14 days, and the interquartile range was 7–41 days. From 2014 onwards, the median time reduced to 12 days, with an interquartile range of 9–15 days.

The enhanced surveillance form (ESF) provides detailed information required for early detection of important changes in the epidemiology of syphilis (see Supplement). Enhanced surveillance data were completed for 541 (65%) of the ES cases notified between July 2016 and June 2018. This proportion is not comparable to the enhanced surveillance data collected with previous case definitions because of the steps undertaken in case confirmation. As it appears, the surveillance system now detects a considerable proportion of ES cases in Ireland and this proportion reflects an increase in the number of cases with enhanced surveillance data available.

The completeness of specific variables on the ESF has also improved which is important for monitoring trends and patterns and identifying at risk groups. Of the notifications with a completed ESF, there was an increase in the completeness of the fields ‘mode of transmission’ (from 85% to 99% complete), ‘HIV status’ (from 79% to 93% complete) and ‘country of birth’ (from 85% to 90% complete).

At the time of the evaluation, the Irish case definition did not include a probable case classification; a further amendment was made in 2019 to include clinical cases diagnosed in clinics for sexually transmitted infections, i.e. cases that are too early to meet the laboratory criteria for notification but that clinicians believe to be ES [15]. The effect of this change will be evaluated on an on-going basis.

There are a number of limitations to the syphilis case definition and the evaluation of the impact of the changes. The case definition does not define re-infections as laboratories in Ireland use their own criteria to identify these cases. This relies on the case being known to the laboratory in question, therefore the proportion of re-infections in Ireland may be underestimated if the individual was previously tested at a different location, although it is likely to be notified as ES by both laboratories. The testing and reporting procedures in place in other jurisdictions will dictate whether this is a concern for other countries, for example, centralised national testing would negate this issue. For logistical reasons, evaluations were confined to the HSE-East area. This area is one of the eight public health areas in Ireland but receives more than 70% of the syphilis notifications nationally. However, the reviews were based on small numbers of cases.

## Conclusion

The surveillance of syphilis is complex owing to the natural history and different stages of infection, complexity of diagnostic testing and potential for re-infection. Over time, Ireland has refined its case definition and we now have a definition that we believe reflects the epidemiology of ES in Ireland accurately and provides timely information for public health action. We welcome the changes introduced in the EU 2018 case definition which addressed some of the problems encountered in Ireland in recent years. However, in light of our experience we suggest considering further refinements, particularly the introduction of a laboratory threshold for notification of RPR of 1:16 and/or positive IgM. This provides timely information for action, while not reducing the sensitivity of the system too much, and for countries where surveillance is driven mainly by laboratory reporting and obtaining clinical details is challenging, it may be a pragmatic solution.

## Supplementary Data

74000 Supplement
