# Supplementary material for: A proposal for changes to the European Union syphilis surveillance case definition using evidence from evaluations in Ireland
Source: Euro Surveill. 2019 Nov 7;24(45):1900311. doi: 10.2807/1560-7917.ES.2019.24.45.1900311 (PMC6852312; doi:10.2807/1560-7917.ES.2019.24.45.1900311)
Supplement: Supplement [file 19-00311_PETTY-SAPHON_Supplement.pdf]

Ireland: Acute Infectious Syphilis Enhanced Surveillance Form

This supplementary material is hosted by *Eurosurveillance* as supporting information alongside the article “A proposal for changes to the European Union syphilis surveillance case definition using evidence from evaluations in Ireland” on behalf of the authors, who remain responsible for the accuracy and appropriateness of the content. The same standards for ethics, copyright, attributions and permissions as for the article apply. Supplements are not edited by *Eurosurveillance* and the journal is not responsible for the maintenance of any links or email addresses provided therein.

Acute Infectious Syphilis Enhanced Form v14 (July 2019)

CONFIDENTIAL

CIDR Event ID:

Section A: Patient Identifiers

Patient Firstname

Patient Surname

Patient Clinic ID

Clinic/Practice Name/Service

Lab specimen ID

Laboratory name

Sex (at birth) Male ☐ Female ☐ Unknown ☐

Date of birth

Gender identity (see note on page 2) Male ☐ Female ☐ Trans male ☐ Trans female ☐ Other ☐

Section B: Case Classification- please choose one (see definitions on page 2)

☐ Confirmed case
- patient meets the clinical & laboratory criteria

☐ Probable case
- patient is symptomatic but does not meet the laboratory criteria

Section C: Patient Information

County of residence (plus postcode)

HSE Area of residence

Country of birth

Ethnic group (see note on page 2)

White
- Irish ☐
- Irish Traveller ☐
- Any other white background ☐

Black or Black Irish
- African ☐
- Any other black background ☐

Asian or Asian Irish
- Chinese ☐
- Any other Asian background ☐

Other
- Roma ☐
- Other, including mixed background ☐

If other ethnic group, please specify ____________________

Section D: Clinical Details

Country of infection:

Probable place of acquisition (e.g. city, sex on premises venue):

Mode of Transmission Heterosexual ☐ MSM (homo/bisexual male) ☐ Other ☐ ____________ Unknown ☐

HIV status Positive ☐ Negative ☐ Unknown ☐ **If HIV positive, year of diagnosis**

Date of diagnosis

	Yes	No	Unk
**Does the patient have symptoms of syphilis?**	☐	☐	☐
**Is the patient pregnant?**	☐	☐	☐
**Was the patient identified via partner notification?**	☐	☐	☐
**Is the patient a commercial sex worker (CSW)**	☐	☐	☐
**Did the patient have contact with a CSW**	☐	☐	☐
**Was patient taking HIV pre-exposure prophylaxis (PrEP) at time of this syphilis diagnosis?**	☐	☐	☐

If yes, please complete section F overleaf

Section E: Form completed by

Completed by:

Date:

Position: Doctor ☐ Nurse ☐ Public health ☐ Health advisor ☐

Comments

For example, service patient was referred to/from

Section F: For cases diagnosed in pregnancy

Patient diagnosed as a result of antenatal screening? Yes ☐ No ☐ Unknown ☐

If yes, gestation at screening ☐☐ /40

History of treated syphilis prior to pregnancy? Yes ☐ No ☐ Unknown ☐

For this pregnancy, date syphilis treatment completed ☐☐/☐☐/☐☐☐☐

Pregnancy outcome Live birth ☐ Stillbirth ☐ Miscarriage ☐ Termination ☐

Gestation at birth ☐☐ /40

Maternity hospital: ____________________

Notes

Sex: Refers to a person's sex at birth

Gender identity: Refers to a person's internal sense of themselves (how they feel inside) as being male, female, transgender or something else. This may be different or the same as a person's assigned sex at birth. Further information and resources can be found at the website of Transgender Equality Network Ireland (www.teni.ie)

Ethnic group: This should be self-reported and is that to which the individual case identifies him or herself. It should not be 'given' by investigator. Categories used as per Census 2016 with the addition of Roma. Further information and resources on collecting ethnic information can be found at https://www.hse.ie/eng/about/ who/primarycare/socialinclusion/ intercultural-health/ethnic-equality-monitoring/

Definitions

Case classification:

A) Possible case
Not applicable

B) Probable case
Any symptomatic person meeting the clinical criteria for early syphilis, as assessed by a specialised STI clinical service (but who does not meet the laboratory criteria)

C) Confirmed case
Any person meeting the clinical criteria for early syphilis, who also meets the laboratory criteria for case confirmation

See http://www.hpsc.ie/a-z/hivstis/sexuallytransmittedinfections/syphilis/casedefinitions/ for the clinical and laboratory criteria.

Please return this completed form to your local Department of Public Health.
See www.hpsc.ie/hpsc/NotifiableDiseases/NotifyingInfectiousDiseases/ for names and contact details

A separate form is available from www.hpsc.ie for congenital cases
